# Treatment with Gold Nanoparticles Using *Cudrania tricuspidata* Root Extract Induced Downregulation of MMP-2/-9 and PLD1 and Inhibited the Invasiveness of Human U87 Glioblastoma Cells

**DOI:** 10.3390/ijms21041282

**Published:** 2020-02-14

**Authors:** Sun Young Park, Zhengwei Cui, Beomjin Kim, Geuntae Park, Young-Whan Choi

**Affiliations:** 1 Bio-IT Fusion Technology Research Institute, Pusan National University, Busan 609-735, Korea; 201210503@pusan.ac.kr; 2 Department of Horticultural Bioscience, Pusan National University, Myrang 627-706, Korea; choejw@pusan.ac.kr; 3 Department of Nanomaterials Engineering, Pusan National University, Busan 609-735, Korea; gtpark@pusan.ac.kr

**Keywords:** CTR-GNPs, *Cudrania tricuspidata*, glioblastoma, MMP-2/-9, PLD1

## Abstract

In this study, we aimed to elucidate the anti-invasive effects of *Cudrania tricuspidata* root-gold nanoparticles (CTR-GNPs) using glioblastoma cells. We demonstrated the rapid synthesis of CTR-GNPs using UV-vis spectra. The surface morphology, crystallinity, reduction, capsulation, and stabilization of CTR-GNPs were analyzed using high resolution transmission electron microscopy (HR-TEM), energy dispersive spectroscopy (EDS), X-ray diffraction (XRD), and Fourier-transform infrared spectroscopy (FT-IR). Furthermore, CTR-GNPs displayed excellent photocatalytic activity as shown by the photo-degradation of methylene blue and rhodamine B. Cell migration and invasion assays with human glioblastoma cells were performed to investigate the anti-invasive effect of CTR-GNPs on U87 cells that were treated with phorbol 12-myristate 13-acetate. The results show that CTR-GNPs can significantly inhibit both basal and phorbol 12-myristate 13-acetate (PMA)-induced migration and invasion ability. Importantly, treatment with CTR-GNPs significantly decreased the levels of metalloproteinase (MMP)-2/-9 and phospholipase D1 (PLD1) and protein but not PLD2, which is involved in the modulation of migration and the invasion of glioblastoma cells. These results present a novel mechanism showing that CTR-GNPs can attenuate the migration and invasion of glioblastoma cells induced by PMA through transcriptional and translational regulation of MMP-2/-9 and PLD1. Taken together, our results suggest that CTR-GNPs might be an excellent therapeutic alternative for wide range of glioblastomas.

## 1. Introduction

Glioblastoma is a devastating condition, and it is the leading cause of brain tumor-associated disability and mortality worldwide. Malignant glioblastoma is characterized by high morbidity and mortality due to metastasis [1]. There has been a substantial increase in the prevalence of glioblastoma, which has led to increased health concerns. In addition, the survival rate of glioblastoma patients is still poor, mainly due to the resistance, reoccurrence, metastasis, and severe treatment side effects [2,3]. Multiple extraneural metastasis of glioblastoma is responsible for the high patient mortality and poses a major challenge in the treatment of glioblastoma due to its complexity and multistep process. Metastasis of glioblastoma is accountable for nearly 90% of all glioblastoma-related deaths due to the spread of primary tumor cells to distant tissues forming secondary tumors that cannot be treated using conventional chemotherapy. Despite tremendous research efforts in recent times, effective, preventative, and disease-modifying treatment options against glioblastoma are not yet available [4,5,6]. The increased incidence of glioblastoma and the problems associated with the development of effective therapeutic alternatives against this disease have prompted researchers to explore nano-medical therapeutic strategies.

The metastasis of glioblastoma cells is a key initiating step in the progression of malignant glioblastoma. Glioblastoma cells promote their mobility, invasion, and metastasis through degradation of the extracellular matrix (ECM) mediated by tumor-associated matrix metalloproteinases (MMPs), such as MMP-2 and MMP-9 [7,8]. MMP-2/-9, which belong to a family of zinc-dependent proteases, play an important role in the degradation of the extracellular matrix during the metastasis of glioblastoma cells by degrading the basement membrane, leading to migration, invasion, and progression of glioblastoma [9,10]. Phorbol 12-myristate 13-acetate (PMA) is one of the most potent inducers of mobility, invasion, and metastasis of glioblastoma cells [11]. Extraneural metastasis of glioblastoma is modulated by various signals from the tumor microenvironment, which elevate the activity of lipases, such as phospholipase D (PLD) [12,13]. Furthermore, the secretion of MMP-2/-9, which is induced by PLD, contributes to the migration and invasion of glioblastoma cells. PLD hydrolyzes the phosphatidylcholine moieties on the cell membrane to generate phosphatidic acid and choline, which can act as intracellular second messengers involved in many physiological events [14,15]. Recently, it has been shown that PLD can inhibit phosphatidic acid production and suppress the activity of MMPs, and this is considered a novel mechanism of action for PLD [16]. PLD activity is elevated extensively by transforming oncogenes, including V-Ras, V-Raf, and V-Src. Therefore, PLD has become one of the major targets for the treatment of a variety of cancers such as glioblastoma, breast cancer, pancreatic cancer, lung cancer, and gastric cancer [17,18,19].

The green synthesis of gold nanoparticles (GNPs) is advantageous over physical and chemical approaches as it is cost effective, maintainable, eco-friendly, dependable, can be easily scaled up, and reduces the production of harmful by-products. The green synthesis of GNPs could also be mediated by the phytochemical composition of reducing, stabilizing, and capping biogenic sources involved in their green synthesis [20]. GNPs have been used for the treatment of a number of diseases due to their biocompatible nature and unique properties, such as conductivity and optical catalytic activity, and because they have better structural characteristics than their bulk counterparts. There is a lot of information available in the literature regarding the molecular targets underlying the anti-tumor properties of biocompatible GNPs [21,22]. GNP-based plants have been experimentally documented to possess medicinal properties as well as various biological properties such as antioxidant, anti-bacterial, anti-inflammatory, and anti-tumor activities [23,24,25]. *Cudrania tricuspidata* is a perennial plant of the Moraceae family, which is mainly distributed in Asian countries such as Korea, China, and Japan. The pharmacological effects of *Cudrania tricuspidata* are due to the presence of a large number of phenolic compounds, including flavonoids, xanthones, alkaloids, and terpenoids [26,27]. More than 100 compounds have been isolated from *Cudrania tricuspidata*, out of which prenylated flavonoids and xanthones are the main constituents that demonstrate a range of pharmacological activities such as antioxidant, anti-microbial, anti-inflammatory, neuroprotective, and hepatoprotective effects [26,28,29,30]. The present study demonstrates that the *Cudrania tricuspidata* root extract is more beneficial for the preparation of GNPs over other conventional methods due to its large number of phytochemicals that are responsible for the reduction, capsulation, and stabilization of GNPs. To the best of our knowledge, this is the first report to elaborate the physicochemical characterization and anti-invasive effects of *Cudrania tricuspidata* root (CTR)-GNPs synthesized from the root extracts of *Maclura tricuspidata* via the inhibition of MMP-2/MMP-9 and PLD activity in human glioblastoma cells.

## 2. Results

### 2.1. Facile and Green Synthesis of CTR-GNPs

**Figure 1:**
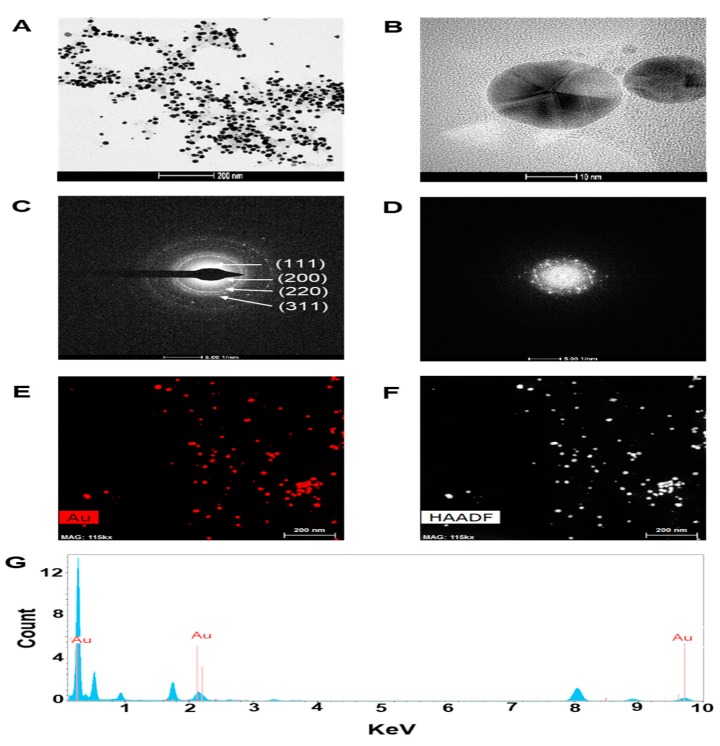
Characterization of *Cudrania tricuspidata* root (CTR)-gold nanoparticles (GNPs). High resolution transmission electron microscopy (HR-TEM) images at (**A**) low magnification (scale bar, 200 nm), (**B**) high magnification (scale bar, 10 nm), (**C**) selected area electron diffraction (SAED) pattern, (**D**) fast Fourier transform (FFT) pattern (**E,F**) high-angle annular dark field (HAADF) image, and (**G**) energy dispersive spectroscopy (EDS) analysis of CTR-GNPs.

In the present study, we assessed the potential of CTR extracts to reduce the gold (III) chloride solution that leads to the formation of GNPs. The addition of CTR extracts to gold (III) chloride solution at room temperature resulted in the formation of CTR-GNPs, which was evident from the color change of the solution from light-yellow to ruby red and from the absorption peak (λmax) seen at 520 nm as a result of surface plasmon resonance of CTR-GNPs. In addition, the resulting CTR-GNPs were further observed using Dynamic light scattering (DLS) to study the hydrodynamic size distribution, zeta potential, and polydispersity index (PDI). We found that the hydrodynamic size distribution, zeta potential, and PDI value of the CTR-GNPs were 89 ± 5.21 nm, −26.93 ± 1.41 mV, and 0.24 ± 0.01, respectively. The surface morphology of CTR-GNPs was explored using high resolution transmission electron microscopy (HR-TEM). Typical HR-TEM micrographs displayed many spherical particles and the CTR-GNPs were found to be an average diameter size of 23.3 ± 3.7 nm (Figure 1A,B). The selected area electron diffraction (SAED) and fast Fourier transform (FFT) pattern of CTR-GNPs also indicated a face-centered cubic crystal structure, which was manifested by bright circular spots (lattice planes of Bragg’s reflection (111), (200), (220), and (311) planes) (Figure 1C,D). Figure 1E shows the representative red-particle image of the GNPs and the corresponding map for the Au atoms. The distribution of the Au atoms in the CTR-GNPs was studied using high-angle annular dark field (HAADF)-TEM (Figure 1F). Energy dispersive spectroscopy (EDS) analysis revealed the surface chemistry of the green synthesized Au-NPs. The results showed three clear peaks of Au at 0.23, 2.16, and 9.73 keV, corresponding to the element present in the CTR-GNPs due to the surface plasmon resonance of gold (Figure 1G).

### 2.2. Physicochemical Characterization of CTR-GNPs

**Figure 2:**
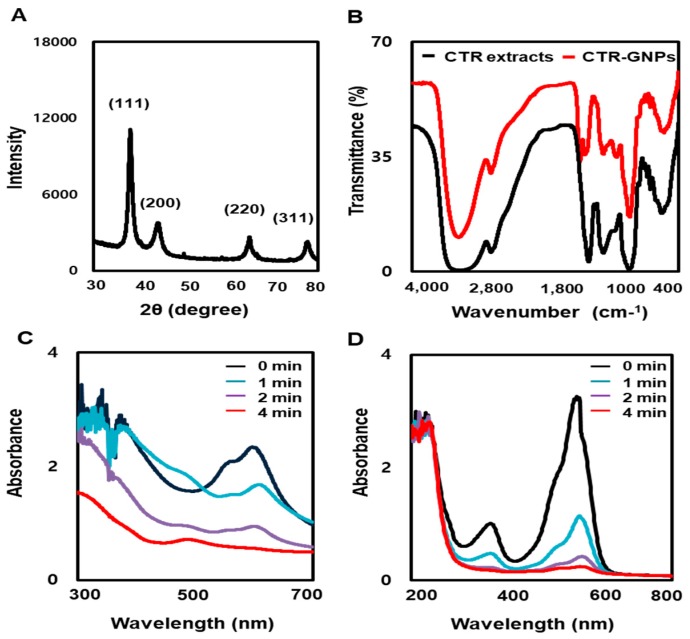
Physicochemical characterization of CTR-GNPs. (**A**) X-ray dispersion (XRD) pattern and (**B**) Fourier-transform infrared spectroscopy (FT-IR) spectra of CTR-GNPs, and UV-visible spectrum of (**C**) methylene blue and (**D**) Rhodamine B after the addition of CTR-GNPs.

To examine the crystallization pattern of the gold particles in the CTR-GNPs, X-ray diffraction (XRD) measurement was recorded, as shown in Figure 2A. The main peaks were obtained at (111), (200), (220), and (311), corresponding to the Bragg’s reflections with 2θ values (30–80°) of 38.21°, 44.39°, 64.73°, and 77.60°, respectively. The peak of the XRD spectrum confirms that the highly purified GNPs are composed of crystalline gold. The GNP-based plants involve biocompatible materials that can act as functionalizing ligands under physiological situations, creating GNPs that are more appropriate for biomedical applications. CTR extracts also provide possible functional groups, which can attach onto the surface of the CTR-GNPs and form a capping and efficient stabilization. In this study, the FT-IR spectra were obtained to acquire further information about the presence of bioactive components in the capping layer of the CTR-GNPs. The FT-IR spectra of the CTR extracts and corresponding CTR-GNPs indicated extensive mutual resemblance. In particular, the prominent peaks of the CTR corresponding CTR-GNPs were characterized by −OH stretching at 3372.01 cm^−1^. −CH stretching was observed at 2930.25 cm^−1^, and we also observed a peak at 1592.45 cm^−1^ corresponding to the C−C stretch of aromatics (Figure 2B). On the basis of these observations, we suggested that the reduction, capsulation, and stabilization of CTR-GNPs were due to the functional groups present in the CTR extract, such as phenols, flavonoids, and proteins. The reduction of methylene blue and rhodamine B was studied using sodium borohydride in the presence of CTR-GNPs and monitored by a UV-Vis spectrophotometer. Characteristic absorption peaks were observed corresponding to pure methylene blue and rhodamine B at 665 and 555 nm, respectively. In the presence of CTR-GNPs as a catalyst, the deep blue (methylene blue) and pink-red (rhodamine B) colors gradually decreased and finally disappeared. In Figure 2C,D, the UV-vis spectra revealed that the peak intensity completely disappeared within 4 min of addition of CTR-GNPs.

### 2.3. CTR-GNPs Inhibit Human U87 Glioblastoma Cell Migration and Invasion

**Figure 3:**
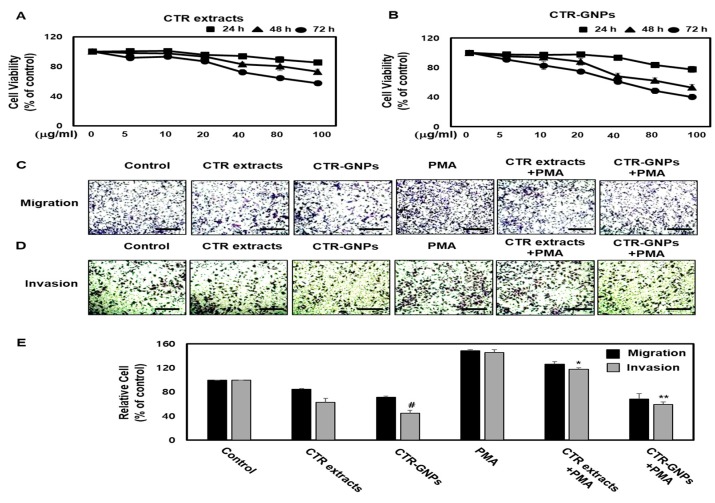
CTR-GNPs attenuate the migration and invasion in basal and phorbol 12-myristate 13-acetate (PMA)-stimulated human U87 glioblastoma cells. (**A**, **B**) Cell viability was detected by cell counting kit-8 (CCK-8) assay. (**C**) Cell migration was detected by the CytoSelect™ 24-Well Cell Migration assay kit (Scale bar, 200 μM). (**D**) Cell invasion was detected by the CytoSelect™ 24-Well Cell invasion assay kit (Scale bar, 200 μM). (**E**) Quantitative analysis of the percentages of migratory and invasive cells. ^#^ *p* < 0.05 compared to the control group (only distilled-water treatment). * *p* < 0.05 and ** *p* < 0.01 compared to the PMA treated group. All data are presented as means ± SEM (*n* = 3).

To investigate the effects of CTR-GNPs on the viability of human U87 glioblastoma cells, the cell counting kit-8 (CCK-8) assay was performed using CTR-GNPs at specific concentrations (0, 5, 10, 20, 40, 80, and 100 µg/mL) for 24, 48, and 72 h. We observed that the cell viability was significantly reduced by the CTR extract and the CTR-GNPs at doses over 80 µg/mL in human U87 glioblastoma cells compared with the control cells. However, treatment with the CTR extract or CTR-GNPs at concentrations of up to 40 µg/mL for 24 h did not significantly alter the cell viability in the human U87 glioblastoma cells. Therefore, for all subsequent experiments, the cells were treated with CTR extract or CTR-GNPs at a dose of 40 µg/mL (Figure 3A,B). We conducted a cell migration assay using U87 cells to assess whether CTR-GNPs could effectively inhibit the motility of the tumor cells. Human U87 glioblastoma cells showed high metastasis ability and the gap repopulated by cells was observed in a cell migration study [11]. As shown in Figure 3C,E, PMA-stimulated U87 cells had significantly increased migration ability over the control group, while treatment with CTR extracts and CTR-GNPs (40 µg/mL) inhibited the migration ability of PMA-stimulated U87 cells. Furthermore, a slight decrease in the migration ability was observed in the U87 cells that were treated with CTR extract or CTR-GNPs alone. Importantly, CTR-GNPs showed a higher inhibitory effect against the motility of U87 cells than the CTR extracts, with or without PMA treatment. The effect on the invasive ability was confirmed using the CytoSelect™ 24-Well Cell Invasion Assay kit. Consistent with the results from the migration assay using U87 cells, we observed an increase in the invasiveness of the cells treated with PMA, which could be inhibited by treatment with the CTR extract and CTR-GNPs. The results of invasion assay also showed that CTR-GMPs significantly decreased the number of invasive cells compared to the CTR extract at an equivalent concentration in PMA stimulated or non-stimulated cells (Figure 3D,E).

### 2.4. CTR-GNPs Inhibit MMP-2/-9 and PLD Activity in Human U87 Glioblastoma Cells

**Figure 4:**
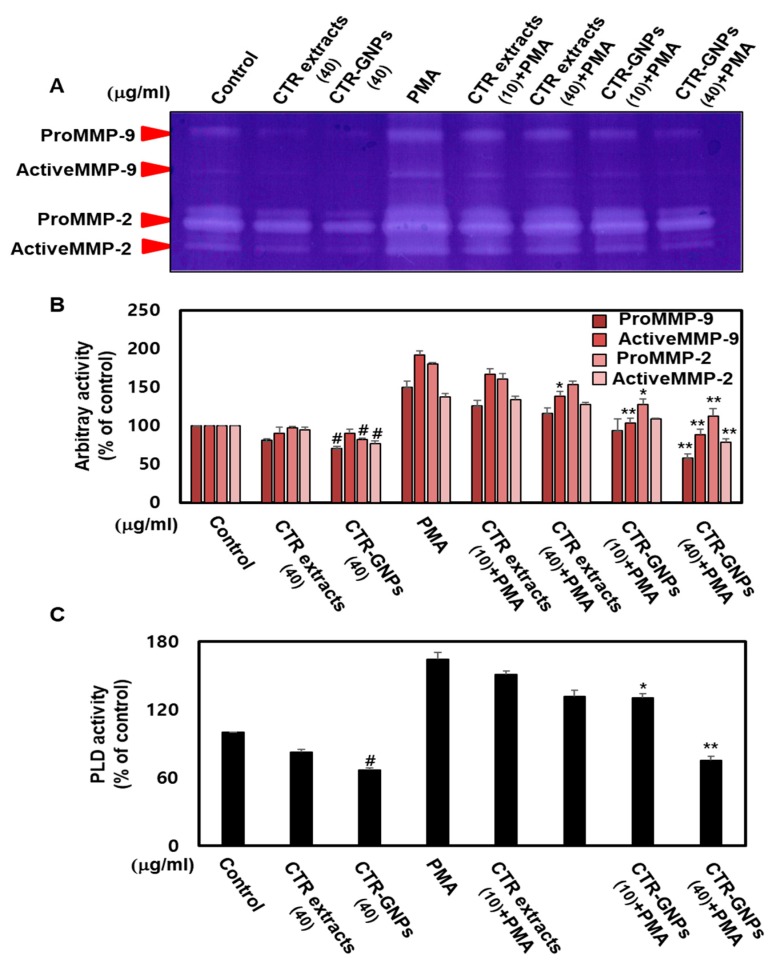
CTR-GNPs inhibited the MMP-2/MMP-9 and PLD1 enzymatic activity in basal and PMA-stimulated human U87 glioblastoma cells. (**A**) The enzymatic activity of metalloproteinase (MMP)-2/MMP-9 was detected by the Zymogram-PAG System kit using the cell culture supernatant. (**B**) Quantitative analysis of the percentage of pro MMP-9, active MMP-9, pro MMP-2, and active MMP-2 enzymatic activity. (**C**) Phospholipase D (PLD) activity was detected by the Amplex Red Phospholipase D Assay Kit. ^#^ *p* < 0.05 compared to the control group (only distilled water treatment). * *p* < 0.05 and ** *p* < 0.01 compared to the PMA group. All data are presented as means ± SEM (*n* = 3).

Accumulating evidence suggests that cell invasion and motility actions are the base characteristics of MMP-2/-9 and PLD [31]. To further understand the anti-invasive activity of CTR-GNPs on human U87 glioblastoma cells, we used gelatinase zymography to confirm the enzymatic activity of MMP-2/-9. The results showed that the enzymatic activity of MMP-2/-9 was significantly inhibited due to CTR-GNPs in PMA-stimulated cells. The CTR-GNPs showed high anti-MMP-2/-9 enzymatic activity as compared to CTR extract in PMA-stimulated groups as well as the non-stimulated group (Figure 4A,B). PLD activity in the tumor cells is one of the most important steps leading to tumor metastasis. Therefore, we further investigated the effect of the CTR extract and CTR-GNPs on the PLD activity of PMA-treated and untreated cells. CTR-GNPs were more effective in inhibiting the PLD activity than CTR, which shows that CTR-GNPs are more highly active than CTR extract (Figure 4C).

### 2.5. CTR-GNPs Inhibit MMP-2/-9 and PLD1 mRNA and Protein Expression in Human U87 Glioblastoma Cells

**Figure 5:**
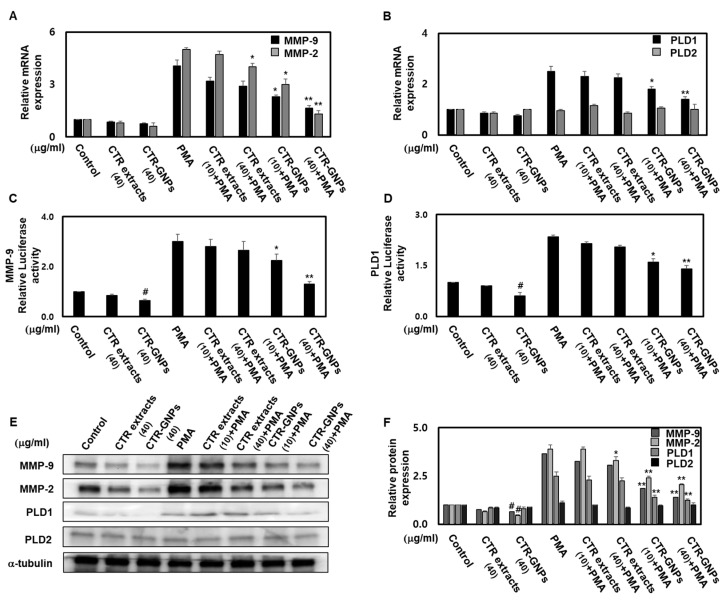
CTR-GNPs downregulate the transcriptional and translational expression of MMP-2/MMP-9 and PLD1 in basal and PMA-stimulated human U87 glioblastoma cells. Real time PCR was carried out to evaluate the mRNA expression of MMP-2/-9 (**A**) and PLD 1/2 (**B**). Cell were transiently transfected with MMP-9 (**C**) and PLD1 (**D**) promoter constructs. Cells were then treated with CTR extract and CTR-GNPs and then treated with PMA. Promoter activity was detected by the Dual Luciferase Reporter assay system kit. (**E**) Western blotting was carried out to evaluate the protein expression of MMP-2/-9 and PLD 1/2. (**F**) Quantitative analysis on the protein expression of MMP-2/-9 and PLD 1/2 was carried out. The control group was treated with only distilled water. * *p* < 0.05 and ** *p* < 0.01 compared to the PMA group. All data are presented as means ± SEM (*n* = 3).

To further investigate the mechanisms of the anti-invasive effect of CTR-GNPs and the roles of MMP-2/-9 and PLD, we studied the effects of CTR extract and CTR-GNPs on the expression of MMP-2/-9 and PLD1/2 in the PMA-treated group and untreated groups. CTR-GNPs decreased the mRNA levels of MMP-2/-9 and PLD1 in human U87 glioblastoma cells treated with or without PMA (Figure 5A,B). CTR-GNPs also inhibited the promoter activity of MMP-9 and PLD1 (Figure 5C,D). We further examined whether CTR-GNPs can suppress the protein expression of MMP-2/-9 and PLD1/2 in human U87 glioblastoma cells. As shown in Figure 5E,F, the results of the Western blotting assay revealed that CTR-GNPs abolished both basal and PMA-induced protein expression of MMP-2/-9 and PLD1/2. Furthermore, treatment with CTR-GNPs significantly downregulated the mRNA, protein expression, and promoter activity of MMP-2/-9 and PLD1/2 compared with that in the CTR extract treated group.

## 3. Discussion

In this study, we provide extensive evidence that the novel CTR-GNPs can attenuate the anti-invasive effects caused by both control and PMA stimulation by downregulating MMP-2/MMP-9 and PLD1. Firstly, we successfully demonstrated the green synthesis of CTR-GNPs for the first time by using CTR extract as a reducing, stabilizing, and capping agent. Secondly, the photocatalytic efficiency of CTR-GNPs was demonstrated by the color changes in methylene blue and rhodamine B. Thirdly, we detected the downregulation of MMP-2/MMP-9 and PLD1 mRNA and protein expression in the CTR-GNP treated group. Finally, we also observed that the CTR-GNPs can significantly inhibit glioblastoma cell migration and invasion in both basal and PMA-stimulated conditions.

Metastases (plural form of metastasis) in glioblastoma are triggered in response to the activation of epithelial mesenchymal transition, local invasion, intravasation, the ability to survive in the bloodstream, extravasation, and the establishment of tumor cells [32]. There have been recent developments in the understanding of the oncogenic role of PLD in tumor progression via viral oncogenes such as v-Ras, V-Src, and V-Fps. The PLD enzyme can hydrolyze the phosphodiester bonds found in the predominant membrane phospholipids, such as phosphatidylcholine, producing phosphatidic acid and free choline, which can regulate cell proliferation, vesicle trafficking, and migration [13]. Two mammalian isozymes of PLD, PLD1 and PLD2, have been identified, and it has been found that they are differentially regulated. For instance, PLD1 plays a key role in the cell transformation induced by H-Ras and is involved in the resistance of cancer cells to chemotherapeutic drugs [33]. Several reports have shown that PLD1 upregulates the expression of MMPs in glioma, melanoma, and fibrosarcoma cells, which, in turn, promotes their migration and invasion [14,17,18]. Based on the above observations, we demonstrated the CTR-GNPs could transcriptionally and translationally inhibit the expression of PLD1 in both basal and PMA-stimulated conditions.

These studies have elucidated the role of MMPs and PLD, indicating that they might possibly contribute to the metastasis of glioblastoma cells. This is shown by the elevated levels of MMPs and PLD in patients with glioblastoma, breast cancer, pancreatic cancer, lung cancer, and gastric cancer [13,19]. MMPs and PLD have emerged as excellent drug targets and can be utilized for the development of alternative therapeutic approaches against cancer [17]. This evidence makes it worth exploring the therapeutic potential of natural MMPs and PLD inhibitors against glioblastoma. CTR-GNPs were shown to exert anti-metastasis potential mainly through their anti-invasive and PLD inhibiting activity, which also resulted in decreased MMP-2/-9 activity. Therefore, CTR-GNPs could be potential therapeutic alternatives against glioblastoma and other MMP-2/-9 and PLD mediated pathologies or conditions.

Due to their beneficial effects, plant-based GNPs have been utilized for the development of nanomedicines for the benefit of humankind [34]. These GNPs are created using the principles of green synthesis to enhance their safety for clinical use. However, we cannot completely undermine the side effects associated with plant-based GNPs as there is always a concern regarding the safety and toxicity of GNP-based therapies, and due to these concerns, plant-based GNPs have merely progressed into the clinical trial stage [25]. *Cudrania tricuspidata* roots, stem, fruits, and leaves have traditionally been used for medicinal purposes to treat conditions such as central nervous system cancer and cardiovascular, kidney, and liver diseases. Interestingly, *Cudrania tricuspidata* roots are reported to be a good source, due to their biological activities related to anti-tumor prevention or therapy [26,35]. To elucidate the potential roles of *Cudrania tricuspidata* roots, stem, fruits, and leaf extracts on green reductants, different parts of *Cudrania tricuspidata* were investigated for total phenol and flavonoid contents. We showed that the root (328.0 ± 16.3 µg GAE/g) extract has the highest phenolic content followed by the stem (233.5 ± 15.8 µg GAE/g) and leaves (131.5 ± 13.9 µg GAE/g), whereas the fruits (77.5 ± 14.0 µg GAE/g) have the lowest phenolic content. The resultant CT-roots-GNPs, CT-stem-GNPs, CT-leaves-GNPs, and CT-fruits-GNPs were further observed by DLS to study the hydrodynamic size distribution, zeta potential, and polydispersity index (PDI). The hydrodynamic size distributions were 89.0 ± 5.21, 88.50 ± 10.16, 91.43 ± 1.87, and 96.60 ± 7.03 nm; the zeta potential values were recorded to be −26.93 ± 1.41, −24.93 ± 0.24, −17.50 ± 0.65, and −24.70 ± 0.21 mV; and the PDI values were recorded to be 0.24 ± 0.01, 0.31 ± 0.02, 0.26 ± 0.02, and 0.32 ± 0.02 for CT-roots-GNPs, CT-stem-GNPs, CT-leaves-GNPs and CT-fruits-GNPs, respectively. However, the inhibitory effect of CT-stem-GNPs, CT-leaves-GNPs, and CT-fruits-GNPs on cell invasion was not significant when compared with the PMA group in the case of U87 cells (data not shown). Future research should focus on developing CTR-GNPs without any side effects. Moreover, future research is warranted to explore and develop CTR-GNPs as inhibitors against the specific isoforms of MMP-2/-9 and PLD that contribute to glioblastoma.

## 4. Materials and Methods

### 4.1. Reagents

Phorbol-2-myristate-13-acetate (PMA), 3-[4,5-dimethylthiazol-2-yl]-2,5-diphenyltetrazoliumbromide (MTT), chloroauric acid (HAuCl_4_·3H_2_O), and other components were obtained from Sigma-Aldrich (Saint Louis, MO, USA). The antibodies against α-tubulin, MMP-9, MMP-2, PLD1, and PLD2 were purchased from Cell Signaling Technology, Inc. (Beverly, MA, USA). All other chemicals were of analytical grade.

### 4.2. Preparation of Cudrania tricuspidata Root (CTR) Extract

The samples of *Cudrania tricuspidata* were collected from Miryang, Gyungnam Province, Korea, in December 2018. The botanical identification was made by Dr. Young Whan Choi (College of Natural Resources and Bioscience, Pusan National University, Korea), and a voucher specimen (No. MT20180011) was deposited at the laboratory of the Natural Products Research Lab, College of Natural Resources and Bioscience, Pusan National University, Korea. The *Cudrania tricuspidata* samples were homogenized into fine particles using an electric mixer (HMF-3100S, Hanil Electric, Seoul, Korea). The dried roots (CTR) of *Cudrania tricuspidata* (30 g) were extracted using distilled water at room temperature, filtered, and concentrated using a rotary vacuum evaporator (Buchi Rotavapor R-144, Buchi Labortechnik, Flawil, Switzerland) to obtain 5.467 g. The extracts were dissolved in distilled water to make a 50 mg/mL stock solution, which was stored at 4 °C and diluted with medium to the desired concentration prior to use.

### 4.3. Synthesis and Physicochemical Characterization of CTR-GNPs

To synthesize the CTR-GNPs, an aqueous solution consisting of 1 mM HAuCl_4_ was mixed with the CTR extract (4 mg/mL). The mixture was rigorously stirred and kept at 25 °C for 15 min. The color change from yellow to violet after 15 min indicated the formation of GNPs. The GNPs were detected using an Evolution™ 300 UV-Vis spectrophotometer (Thermo Fisher Scientific, Tulsa, OK, USA). Dynamic light scattering and zeta-potential analysis were performed on all samples, and the data were recorded using Zetasizer Nano ZS90 (Malvern Instruments, Malvern, UK). X-ray diffraction (XRD) measurements were performed using an X’Pert^3^ Powder X-ray Diffractometer (Malvern Panalytical, Malvern, UK) operating at a scanning range of 30 to 80°, a voltage of 40 kV, and a current of 30 mA. Fourier-transform infrared spectroscopy (FT-IR) measurements were carried out on a Perkin Elmer Spectrum GX FT-IR spectrophotometer operating using KBr pellets in the range of 4000 to 400 cm^−1^. The surface morphology, crystallinity, and chemical composition of the CTR-GNPs were examined using high resolution transmission electron microscopy (HR-TEM), selected area electron diffraction (SAED), fast Fourier transform (FFT), high-angle annular dark field (HAADF), and energy dispersive spectroscopy (EDS) measurements, which were performed using a Thermo Scientific (FEI) Talos F200X G2 TEM instrument.

### 4.4. Photocatalytic Activities of CTR-GNPs

The photocatalytic activity of CTR-GNPs was evaluated by observing the degradation of methylene blue and rhodamine B dye. In brief, CTR-GNPs were added to a solution containing methylene blue (0.8 mM) and rhodamine B (0.05 mM), and then, ice cold sodium borohydride (0.06 M) solution was added. The degradation of the dye was monitored using an Evolution™ 300 UV-Vis spectrophotometer (Thermo Fisher Scientific, Tulsa, OK, USA) in the range of 300–800 nm with regular intervals (1 min).

### 4.5. Cell Culture and Cell Viability

Human U87 glioblastoma cell lines were purchased from American Type Culture Collection (ATCC, Manassas, VA, USA). They were cultured with Eagle’s minimum essential medium (MEM) (Gibco; Thermo Fisher Scientific, OK, USA) containing 10% Fetal bovine serum (FBS, Gibco; Thermo Fisher Scientific, OK, USA) and 1% penicillin/streptomycin (Gibco; Thermo Fisher Scientific, Tulsa, OK, USA) at 37 °C with 5% CO_2_. A cell viability assay was performed using the CCK-8 assay (Sigma-Aldrich, St. Louis, MO, USA) in a solution (5 g/L, dissolved in PBS) according to the manufacturer’s instructions. The optical density value was determined using a FLUOstar^®^ Omega Plate Reader (BMG Labtech, Ortenberg, Germany) at 450 nm.

### 4.6. Cell Transwell Migration and Invasion Assay

The effects of the CTR extract or CTR-GNPs on the migration of U87 cells was evaluated using the CytoSelect™ 24-Well Cell Migration assay (8 μm, Cell Biolabs, San Diego, CA, USA) according to the manufacturer’s instructions. Similarly, the U87 cells that migrated to the bottom surface were stained with cell stain solution for visualization and then extracted using extraction solution. The OD at 560 nm was measured using a FLUOstar^®^ Omega Plate Reader (BMG Labtech). The cell invasion assays were performed using the CytoSelect™ 24-Well Cell Invasion Assay (Basement Membrane, Cell Biolabs, San Diego, CA, USA) according to the manufacturer’s instructions. Similarly, U87 cells that penetrated to the bottom surface were stained using cell stain solution for visualization, extracted using extraction solution, and the OD at 560 nm was measured using a FLUOstar^®^ Omega Plate Reader (BMG Labtech).

### 4.7. Gelatinase Zymography

To evaluate the enzymatic activity of MMP-2/-9, the U87 cells were seeded onto 6-well plates, incubated for 24 h to allow attachment, and then, treated with equivalent concentrations of CTR extracts or CTR-GNPs at 50 ng/mL of PMA for a further 24 h. The cell culture media was mixed with the Zymogram sample buffer from the Zymogram-PAG System kit (Komabiotech, Seoul, Korea) according to the manufacturer’s instructions. After electrophoresis, the gels were washed with Zymogram renaturing buffer and then incubated overnight at 37 °C with the Zymogram developing Buffer. Then, the gels were stained and destained with Coomassie Brilliant Blue R-250 Staining Solutions Kit (Bio-rad, Richmond, CA, USA).

### 4.8. Determination of PLD Activity

PLD activity was quantified using the Amplex Red Phospholipase D Assay Kit (Molecular Probes) according to the manufacturer’s protocol. In brief, the cultured U87 cells were pre-treated with the CTR extract or CTR-GNPs for 1 h before an additional treatment with PMA for 24 h, washed with PBS, and lysed using three freeze–thaw cycles in ice-cold lysis solution. Lysates were mixed with an equal amount of the Amplex Red reaction buffer, and the PLD activity was determined using the FLUOstar^®^ Omega Plate Reader (BMG Labtech). The data for the PLD activity were normalized with respect to the protein concentration of the cell lysate.

### 4.9. Total RNA Extraction and Quantitative Real Time PCR Analysis

Total RNA was isolated from U87 cells using the RNeasy Mini kit (QIAGEN, Hilden, Germany), and cDNA templates were generated by reverse transcription using high-capacity cDNA reverse transcription kit (Thermo Fisher Scientific, Tulsa, OK, USA). Quantitative Real Time PCR (qRT-PCR) was performed using SYBR Green qPCR master mixes (Thermo Fisher Scientific, Tulsa, OK, USA). Real time PCR assays were performed according to the manufacturer’s instructions.

### 4.10. Protein Preparation and Western Blotting Analysis

U87 cells were harvested and split using M-PER mammalian protein extraction reagent (Thermo Fisher Scientific, Tulsa, OK, USA) according to the manufacturer’s instructions. The protein concentration was determined using the Bio-Rad protein assay kit (Bio-rad, California, USA). The proteins in the cell lysates were separated using 7–12% SDS-PAGE gels, and then, they were electrotransferred onto a PVDF membrane (Amersham Biosciences, Piscataway, NJ, USA). The protein bands were detected using the enhanced Pierce ECL Western Blotting Substrate (Thermo Fisher Scientific, Oklahoma, USA) and quantified as the ratio to the α-tubulin levels. The quantification was performed using an ImageQuant 350 analyzer (Amersham Biosciences).

### 4.11. Statistical Analysis

The data analyses were done with Statistical Package for the Social Sciences software (version 17.0). Student’s *t* test and one-way analysis of variance (ANOVA) were used to evaluate the differences among groups. *P*-values of < 0.01 or < 0.05 were considered to indicate statistically significant differences.

## 5. Conclusions

Our study illustrates that the green synthesis of GNPs with *Cudrania tricuspidata* enhances the anti-invasive effects when compared to using *Cudrania tricuspidata* root extracts alone, possibly because of the presence of the functional groups from the CTR extract that act as reducing, stabilizing, and capping agents. Our results also demonstrate that CTR-GNPs show more effective inhibition than CTR extract on cell migration and invasion via the downregulation of MMP-9 and PLD activity and expression. Based on the results of this study, we conclude that CTR-GNPs can potentially inhibit metastasis and assist in the treatment of glioblastoma.
